# The *Green Surgery* report: a guide to reducing the environmental impact of surgical care, but will it be implemented?

**DOI:** 10.1308/rcsann.2024.0005

**Published:** 2024-04-29

**Authors:** M Bhutta, C Rizan

**Affiliations:** Brighton and Sussex Medical School, UK

In recent years, the agenda to reduce the environmental impact of surgical care has been met with enthusiasm but it has yet to achieve the scale and nature of the change we need. At least in part, that is perhaps because many surgical teams are still not sure what to do. Historically, attempts to decarbonise the UK health system were dominated by strategy to improve the efficiency of health estates and facilities.^1^ However, building energy, water and waste account for only 15% of the carbon footprint of the National Health Service (NHS).^2^ There is increasing recognition of the need for clinical leadership on this agenda and the much larger effects this could have, for example through transforming care pathways or reducing emissions from the medicines and products used to deliver care.^3^

Surgery is an area calling out for transformation because it is such a large contributor. Our analysis estimates the annual carbon footprint of surgical care in the UK at 5.7 million tonnes of carbon dioxide equivalents (CO_2_e),^4^ with operating theatres particularly resource-intensive (typically generating a quarter of hospital waste).^5^ Offsetting that would mean planting a forest three times the size of Greater London.

November 2023 saw the release of *Green Surgery: Reducing the Environmental Impact of Surgical Care* (Figure 1), a report funded by the Health Foundation and produced in collaboration by the UK Health Alliance on Climate Change, the Centre for Sustainable Healthcare, and our team at Brighton and Sussex Medical School.^4^ The report provides a compendium of current evidence and case studies on how to minimise the environmental harm of surgical care, including through disease prevention, changing care pathways, better design and use of operating theatres, and changing practice in anaesthesia.

**Figure 1:**
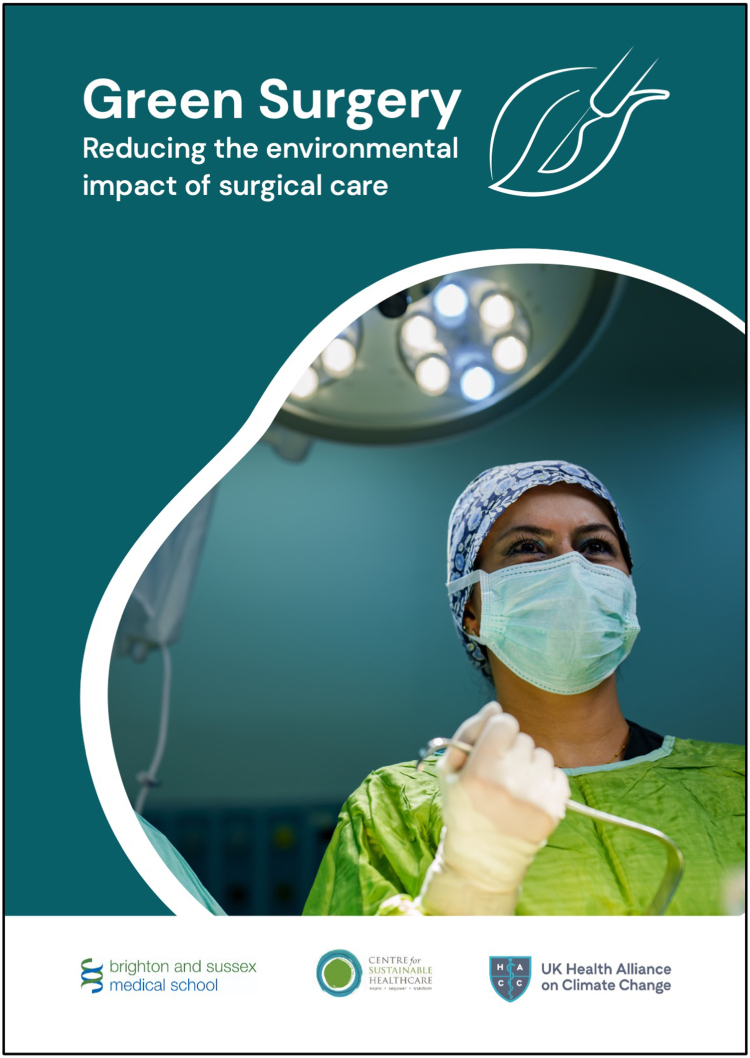
Green Surgery report,^4^ available from https://ukhealthalliance.org/sustainable-healthcare/green-surgery-report/

*Green Surgery* is the first report of its kind by any medical specialty and badged by many organisations, including all four UK surgical colleges.^4^ It is intended primarily as a tool for surgical teams (surgeons, anaesthetists, nurses and operating department practitioners) but also outlines actions and recommendations for other stakeholders, including hospital managers, government, academics, industry and providers of services allied to surgical care.

## Implementing green surgery

To what extent the *Green Surgery* report will lead to real-world change remains to be seen. We may be encouraged by our previous survey in 2021, which showed that most surgeons were willing to make changes in their practice to support environmental sustainability but asked for greater leadership and guidance from national bodies.^6^ Nevertheless, we may be discouraged by a 2022 survey of UK perioperative staff, which found that only a quarter of departments had implemented any education or actions towards environmental sustainability.^7^

The *Green Surgery* report is best viewed as an evidence base and tool for action but will need ongoing commitment to support implementation, and an exploration of the barriers and incentives to achieving that. Previous studies show that sustained change in health systems relies on a number of factors, including distributed leadership and collaborative relationships (i.e. people in an organisation coming together and supporting each other to drive and maintain change).^8^ That must be underpinned by a belief in the cause, namely that climate change is important and that we should do our bit to mitigate harm. The recent shift in UK public attitudes towards climate change^9^ suggests that we are on a good footing.

Some changes occur quicker than others, and so we also need some granularity in understanding what makes some change seem easy and some more difficult. How can we incentivise progress and remove barriers? In November 2022, we saw the launch of the Intercollegiate Green Theatre Checklist, a summary of evidence-based actions to reduce environmental impact in the operating theatre.^10^ A study of general surgery at Bradford Royal Infirmary in 2023 reported good performance against some areas of the checklist (such as preferencing reusable textiles and avoiding unnecessary urethral catheterisation) but performance was less satisfactory for others (such as using alcohol for surgical scrubbing and minimising use of non-sterile gloves).^11^ The reasons behind variation in uptake may be behavioural, cultural or logistical and are deserving of further analysis.

We should also recognise challenges common to instigating change in most health systems: disjointed providers of care, complex systems and long-established cultures of clinical practice, which together give rise to system inertia. In addition, healthcare staff are overstretched in the workplace and so we need to do our best to make the environmentally sustainable choice an easy choice. This includes developing national infrastructure that supports remote care or self-care for patients^12^ (rather than a scattergun use of systems and external providers that may fail),^13^ with acknowledgement that remote care is not a panacea. (We all recognise the value of in-person consultation for clinical examination, and for enabling depth and subtlety in the doctor–patient relationship.)

Furthermore, we need to improve the availability of surgical products that are reusable while at the same time matching this with expanded resources for sterilisation or decontamination to assist in accomplishing that change. We also need to provide protected staff time to support and enable this transition to greener approaches.

Perhaps relying on goodwill and a few committed individuals is not enough. In England, the inspector of health providers, the Care Quality Commission, has announced that it is exploring the introduction of mandatory reporting on environmental performance as an added marker of good healthcare governance.^14^

## The economics of greening surgical care

The elephant in the room is how we are going to fund this change, in the middle of a financial and human resource crisis in the NHS^15^ (mirrored in other health systems around the world). We should embrace this elephant because although greening healthcare may require upfront investment of time and money, evidence to date (chronicled in the *Green Surgery* report)^4^ shows that in fact, it almost always saves money.

And this topic deserves more than case studies: we need a fundamental reconsideration of our financial models of purchasing. In England, NHS Supply Chain buys close to a staggering 600,000 different medical products (Heidi Barnard, head of sustainability, NHS Supply Chain, personal communication), with a strategic focus on getting value for money. We should take a step back and think about what the NHS is actually buying, and here, two green strategies come to mind. The first is that the NHS should focus on preferable purchasing of reusable products^16^ (where clinically safe to do so), which will reduce both the volume of products it buys and their associated environmental harm. A review from 2023 found that the carbon footprint of reusable medical goods is 38–56% smaller than that for single-use equivalents, including decontamination or sterilisation of those products.^17^

Second, the NHS should increase its use of servitisation (i.e. where we buy services rather than products). This drives suppliers to create products designed to last and be repaired because if the NHS pays a fixed price for the service, it is the supplier’s bottom line that suffers if they need to replace that product too soon. Evidence from non-healthcare contexts suggests that for every 1% increase in servitisation, carbon emissions embodied in manufacturing export trade reduce by 1–2%.^18^

Perhaps the current exemplar of servitisation in the NHS is that of surgical drapes and gowns, where reusable versions supplied on a service contract decrease carbon 2–3-fold compared with the disposable equivalent.^19^ Nevertheless, at present, two-thirds of gowns and three-quarters of drapes in England and Wales are disposable (Roberta Charlett, head of marketing, Elis UK, personal communication), and in Scotland, all are disposable (Wendy Rayner, head of NHS Scotland Circular Economy Programme, personal communication).

The persistence of disposable products may be driven by convenience or by fear of infection and some suppliers will play on those fears. The lead author (MB) was once removed from an industry-sponsored webinar on surgical drapes and gowns because he wanted to make the point that evidence^20^ and standards^21^ show that reusable drapes and gowns are entirely safe. He has also been harassed at a conference by a supplier of single-use endoscopes, for pointing out their unfounded marketing on infection risk and environmental benefit.^22^

Suppliers of reusable products may welcome the shift to economic servitisation as a means to support them in producing and maintaining high-quality products. Suppliers of single-use products, on the other hand, more likely see the green agenda as a threat to their economic business model.

## Shared leadership

Climate change is nothing new. In 1938, the amateur scientist Guy Callendar showed that global temperatures were rising and suggested that this was because of the burning of fossil fuels.^23^ Since the 1970s, fossil fuel companies have tried purposefully to distort the science on the effects on the climate from the burning of fossil fuels.^24^

Humanity has been late to recognise and respond to this threat, and many of the financial and logistical structures we have built over the past century are fundamentally destructive to our planet. Let us show individual and collective leadership to rethink our journey, to build something sustainable. Something better.

## References

[1] NHS England. Saving carbon, improving health: update – NHS carbon reduction strategy. https://www.england.nhs.uk/greenernhs/wp-content/uploads/sites/51/2021/02/NHS-Carbon-Reduction-Strategy-2009.pdf (cited February 2024).

[2] Tennison I, Roschnik S, Ashby B *et al*. Health care’s response to climate change: a carbon footprint assessment of the NHS in England. *Lancet Planet Health* 2021; **5**: e84–e92. 33581070 10.1016/S2542-5196(20)30271-0 PMC7887664

[3] Health Foundation. Net zero care: what will it take? https://www.health.org.uk/publications/long-reads/net-zero-care-what-will-it-take (cited February 2024).

[4] Brighton and Sussex Medical School, Centre for Sustainable Healthcare, UK Health Alliance on Climate Change. *Green Surgery: Reducing the Environmental Impact of Surgical Care*. London: UKHACC; 2023.

[5] Rizan C, Steinbach I, Nicholson R *et al*. The carbon footprint of surgical operations: a systematic review. *Ann Surg* 2020; **272**: 986–995. 32516230 10.1097/SLA.0000000000003951

[6] Harris H, Bhutta MF, Rizan C. A survey of UK and Irish surgeons’ attitudes, behaviours and barriers to change for environmental sustainability. *Ann R Coll Surg Engl* 2021; **103**: 725–729. 34719956 10.1308/rcsann.2021.0271 PMC10335270

[7] Gadi N, Lam K, Acharya A *et al*. Perceptions and priorities of perioperative staff and the public for sustainable surgery: a validated questionnaire study. *Ann Med Surg* 2023; **85**: 2400–2408. 10.1097/MS9.0000000000000289 PMC10289730 37363477

[8] Willis CD, Saul J, Bevan H *et al*. Sustaining organizational culture change in health systems. *J Health Organ Manag* 2016; **30**: 2–30. 26964847 10.1108/JHOM-07-2014-0117

[9] Liu T, Shryane N, Elliot M. Attitudes to climate change risk: classification of and transitions in the UK population between 2012 and 2020. *Humanit Soc Sci Commun* 2022; **9**: 279. 35996468 10.1057/s41599-022-01287-1 PMC9386649

[10] Royal College of Surgeons of Edinburgh. Green Theatre Checklist. https://www.rcsed.ac.uk/professional-support-development-resources/environmental-sustainability-and-surgery/green-theatre-checklist (cited February 2024).

[11] Westwood E, Walshaw J, Boag K *et al*. Time for change: compliance with RCS green theatre checklist – facilitators and barriers on the journey to net zero. *Front Surg* 2023; **10**: 1260301. 37942001 10.3389/fsurg.2023.1260301 PMC10628494

[12] Greenhalgh T, Rosen R, Shaw SE *et al*. Planning and evaluating remote consultation services: a new conceptual framework incorporating complexity and practical ethics. *Front Digit Health* 2021; **3**: 726095. 34713199 10.3389/fdgth.2021.726095 PMC8521880

[13] Wired. The fall of Babylon is a warning for AI unicorns. https://www.wired.co.uk/article/babylon-health-warning-ai-unicorns (cited February 2024).

[14] Care Quality Commission. Environmental sustainability – sustainable development. https://www.cqc.org.uk/assessment/quality-statements/well-led/environmental-sustainability (cited February 2024).

[15] Institute for Government. *The NHS Crisis: Does the Sunak Government Have a Plan?* London: IfG; 2023.

[16] Bhutta MF. Our over-reliance on single-use equipment in the operating theatre is misguided, irrational and harming our planet. *Ann R Coll Surg Engl* 2021; **103**: 709–712. 34719955 10.1308/rcsann.2021.0297 PMC10335235

[17] Keil M, Viere T, Helms K, Rogowski W. The impact of switching from single-use to reusable healthcare products: a transparency checklist and systematic review of life-cycle assessments. *Eur J Public Health* 2023; **33**: 55–63. 10.1093/eurpub/ckac174 PMC9898010 36433787

[18] Li X, Wang X, Zhang Y, Miao X. Spatial differences in emission reduction effect of servitization of manufacturing industry export in China. *Emerg Mark Financ Trade* 2021; **57**: 2331–2355.

[19] Overcash M. A comparison of reusable and disposable perioperative textiles: sustainability state-of-the-art 2012. *Anesth Analg* 2012; **114**: 1055–1066. 22492184 10.1213/ANE.0b013e31824d9cc3

[20] Kieser DC, Wyatt MC, Beswick A *et al*. Does the type of surgical drape (disposable versus non-disposable) affect the risk of subsequent surgical site infection? *J Orthop* 2018; **15**: 566–570. 29881195 10.1016/j.jor.2018.05.015 PMC5990293

[21] NHS England. Health Technical Memorandum 01-04: Decontamination of linen for health and social care. https://www.england.nhs.uk/publication/decontamination-of-linen-for-health-and-social-care-htm-01-04 (cited February 2024).

[22] Rizan C, Bhutta MF. Re: The carbon footprint of single-use flexible cystoscopes compared with reusable cystoscopes: methodological flaws led to the erroneous conclusion that single-use is ‘better’. *J Endourol* 2022; **36**: 1466–1467. 35848502 10.1089/end.2022.0482

[23] Callendar GS. The artificial production of carbon dioxide and its influence of temperature. *Q J R Meteorol Soc* 1938; **64**: 223–240.

[24] Supran G, Rahmstorf S, Oreskes N. Assessing ExxonMobil’s global warming projections. *Science* 2023; **379**: eabk0063. 36634176 10.1126/science.abk0063

